# Human Blastoid: A Next-Generation Model for Reproductive Medicine?

**DOI:** 10.3390/biology14101439

**Published:** 2025-10-18

**Authors:** Anfisa S. Ryabchenko, Vepa K. Abdyev, Ekaterina A. Vorotelyak, Andrey V. Vasiliev

**Affiliations:** Koltzov Institute of Developmental Biology of the Russian Academy of Sciences, Moscow 119334, Russia

**Keywords:** blastoid, pluripotent stem cells, blastocyst-like structures, self-organization, implantation model, synthetic embryology, ART (assisted reproductive technology)

## Abstract

**Simple Summary:**

Early human embryogenesis remains poorly investigated. At the same time, the problems of studying early human development are relevant due to the large number of unsuccessful pregnancies and early embryonic mortality. However, the use of human embryos in studies has ethical and legal restrictions, whereas animal models have differences in the mechanisms of early development, which reduces their suitability. As an alternative, models of embryos from pluripotent stem cells, which are useful for imitating the early stages of human development, have been proposed. Such models were called blastoids. Using them may become beneficial not only for studying early human development but also for developing new approaches in the field of assisted reproductive technologies, in particular, for identifying pathologies associated with conception and the first weeks of pregnancy.

**Abstract:**

Human early embryogenesis remains unexplored due to limited access to human embryos for research purposes. Meanwhile, the number of natural early pregnancy terminations remains significant, and solving the problem requires a deep understanding of the developmental mechanisms of this period. Although assisted reproductive technologies (ART) utilize up-to-date approaches in culturing human embryos *in vitro*, characterization of the embryos is still based on visual evaluation and subjective assessment. In addition, embryonic development in animal models, such as rodents and cattle, correlates poorly with human embryonic development. Synthetic embryology presents a promising new approach for studying human embryos involving the creation of embryos without the use of haploid germ cells. Instead, diploid pluripotent stem cells (PSCs) in a given state of pluripotency, which is maintained under conditions of induction and/or inhibition of certain signaling pathways, are used. Synthetic embryo systems (SES) may become a successful alternative model for studying fundamental processes of human early preimplantation embryogenesis, exploring new methods of objective embryo qualification, and personalized approaches in ART. However, the question of whether SES models can be considered as full-fledged mimics of the embryo remains open. This review examines human blastocyst-like structures known as blastoids. It discusses their use as models, as well as the parameters that need to be modified to more accurately simulate the human blastocyst.

## 1. Introduction

**Figure 1:**
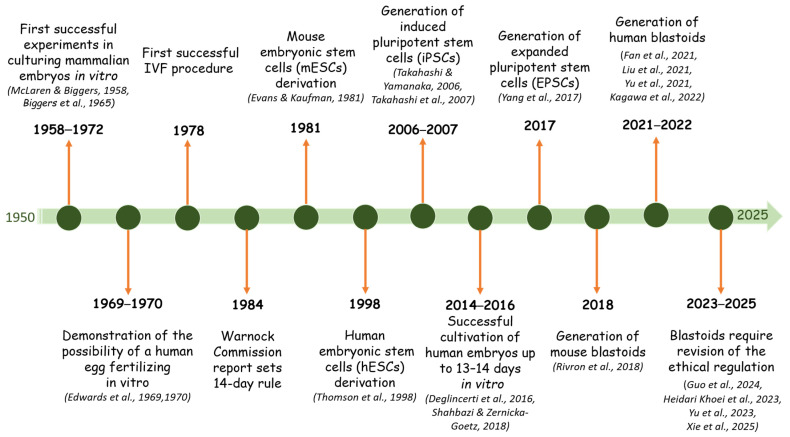
Prerequisites and steps of development of synthetic embryology and blastoid research [1,2,3,4,5,6,7,8,9,10,11,12,13,14,15,16,17,18,19,20,21,22,23,24].

Synthetic embryology is a branch of developmental biology devoted to the creation of models imitating the early development of mammals from pluripotent stem cells. This is a cross-disciplinary scientific field that emerged in the 21st century at the intersection of experimental embryology, developmental biology, genetic engineering, and stem cell biology. The preconditions for the emergence of this scientific field arose much earlier, when it became possible to cultivate mammalian embryos *in vitro* [1,2] (Figure 1). Synthetic embryology is a branch of embryology that focuses on creating models based on pluripotent stem cells that imitate the early development of mammals. By comparison, classical embryology studies the development of an organism from a single cell, a totipotent zygote, which is formed as a result of the fertilization of a haploid egg by a sperm [3,4,5]. The totipotent zygote undergoes maturation through cleavage, activation of zygotic genes, and morphogenesis of cell layers [3].

The human embryo at the 8-cell stage initiates a gene regulation program to activate epithelialization and compaction, which is a crucial moment for self-organization and further maturation. The separation of the polar trophectoderm cell lineages and the nonpolar pluripotent cells that will form the inner cell mass (ICM) of the blastocyst, consisting of the NANOG^+^ epiblast and NANOG^−^ hypoblast, occurs via morphogenetic processes. As a result, the human blastocyst comprises three cell types: the embryonic part (epiblast), the extraembryonic part (trophectoderm), and the hypoblast.

Embryo-like structures, which are the subjects of synthetic embryology, are created from diploid pluripotent stem cells (PSC) in a given state of pluripotency maintained based on certain conditions of induction and/or inhibition of signal pathways and/or gene engineering methods [25,26,27,28,29]. The co-culture of trophoblast stem cells (TSC) and PSC, which form embryo-like structures in three-dimensional conditions, is often required for the complete development of the “synthetic” embryo [30]. The use of *in vitro* generated synthetic embryo systems (SES) opens up incredible opportunities for fundamental research of human embryonic development, modeling of inherited diseases, regenerative medicine, and drug screening [31,32,33,34].

## 2. Difficulties in Studying Human Embryonic Development

Early embryonic development is a dynamic process accomplished by morphogenetic mechanisms that operate via the organized time-bound expression of specific genes and the correct spatial distribution of agonistic protein products of these genes with the antagonistic effects of counteracting morphogens. Currently, early human embryogenesis is not available for study due to ethical considerations and legal restrictions [8]. Embryos obtained as a result of *in vitro* fertilization (IVF) that have been assessed as unsuitable for transfer into the patient’s uterus and/or after the patient’s refusal with consent for research purposes are utilized for studies of early human embryogenesis. However, there is a “14-day rule” that prohibits culturing human embryos for more than 14 days or after the formation of the primitive streak [34,35,36,37,38]. The formation of the primitive streak is a key stage in early embryonic development occurring approximately 14 days after fertilization. This process marks the beginning of gastrulation and the establishment of the embryo’s spatial organization. This is used as a criterion for establishing the individuality of the embryo, although the exact biological boundary remains a subject of debate [39]. The issue of culturing human embryos was first raised in 1978 after the first successful IVF procedure [6,7]. This led to public and political debates and ultimately resulting in the proposal to introduce a 14-day limit on culturing human embryos *in vitro* [8]. Unfortunately, it was rarely possible to culture embryos for more than 7–9 days, so the 14-day rule was well within the technical and methodological capabilities of researchers [40]. However, successful culturing of human embryos for more than 14 days was subsequently achieved, and this scientific breakthrough required a revision of the 14-day rule [13,14,41]. Following numerous appeals to the International Society for Stem Cell Research (ISSCR), the Society published new regulations for embryo culturing in 2021 [42]. According to the ISSCR, such research can be carried out under strict scientific rationale, using a limited number of embryos and under the supervision of specialized committees. The decision on terminating the experiment is made individually based on ethical assessments and the scientific goals achieved [42].

The restriction on studying human embryos after 14 days of development established in several countries contributes to the incompleteness of the fundamental understanding of the molecular and cellular processes of early embryogenesis. This is of particular importance in light of the data on the high frequency of pregnancy terminations at preclinical stages, reportedly reaching 10–70% by the end of the second week of gestation [43,44]. The limited time of study, along with the lack of adequate models that reproduce critical events before and during implantation *in vitro* and *in vivo*, significantly complicates the establishment of causal relationships between early embryogenesis disorders and pregnancy outcome. In the field of ART, new approaches are being developed to solve the problem of miscarriage at the preimplantation stage. However, the effectiveness of such strategies is limited not only by available technological solutions but also by the lack of fundamental knowledge about the mechanisms and regulation of early development of the human embryo.

Gaps in our understanding of early embryogenesis extend beyond implantation processes to earlier stages, starting with the movements of the oocyte and the embryo in the fallopian tubes during conception, which is a critical physiological event [45]. At this point, the embryo undergoes numerous important biophysical and biochemical interactions that contribute to its proper and efficient development at subsequent stages [45]. Although previous studies have shown that interactions with the oviduct are not essential for embryonic development, processes like oviductal fluid production, ciliary beating, and smooth muscle peristalsis as well as embryotrophins secreted by the embryo itself are crucial for successful embryonic development and subsequent delivery [46,47,48]. In addition, the *in vitro* cultured embryo develops in static conditions and does not interact with the mother’s body, which can lead to a deficiency of several critical molecular and biochemical signals that determine the success of preimplantation development [49,50].

The interaction of the embryo with the endometrium during the preimplantation period is mediated by a complex network of signaling molecules that ensure the synchronization of cell proliferation, differentiation, immunomodulation, and angiogenesis. One of the key elements of this network is leukemia inhibitory factor (LIF), a cytokine expressed by glandular cells of the endometrium within the “implantation window” and inducing the expression of adhesion molecules, such as integrins, on the surface of the trophoblast, which is critical for its attachment to the endometrium [51,52]. Insulin-like growth factors (IGF-I, IGF-II) play a bidirectional regulatory role by stimulating the mitotic activity of both trophoblast and endometrial cells and modulating metabolic activity [53]. In addition, IGFs enhance the expression of vascular endothelial growth factor (VEGF), which promotes angiogenesis and the formation of a microvascular network in the implantation zone [54]. VEGF is a central mediator of angiogenesis regulating the growth and permeability of endometrial vessels thereby ensuring the delivery of oxygen and nutrients to the developing embryo [55,56]. In addition to angiogenic signals, immunomodulatory factors play an important role, in particular, colony-stimulating factor-1 (CSF-1), which regulates the activation and polarization of macrophages in the endometrium creating a local immune environment favorable for implantation and stimulates trophoblast proliferation [57]. Heparin-binding epidermal growth factor (HB-EGF) is a key mediator in establishing direct intercellular contact between the trophoblast and endometrial epithelial cells. It increases endometrial receptivity by enhancing the expression of molecules involved in adhesion and intercellular interactions [58,59]. This regulatory network is augmented by preimplantation factor (PIF), a peptide secreted by the trophoblast from the first days after fertilization. PIF modulates the expression of genes associated with cell adhesion and survival, has an anti-apoptotic effect, and helps to maintain local maternal immune tolerance to the embryo [60].

Under physiological conditions, these signaling molecules form an interconnected network in which the endometrium and the embryo exchange biochemical signals, which positively influence cell proliferation, differentiation, immunological tolerance, and angiogenesis. During *in vitro* cultivation, the lack of a full-fledged bidirectional exchange of signals with the mother’s body disrupts the fine spatiotemporal coordination of these processes, which can affect morphogenesis and reduce the likelihood of successful implantation.

Another limitation is that current commercial human embryo culture media are primarily developed using animal models and/or somatic cells [61,62]. There are many differences in early development between humans and other mammals, such as mice, cattle, and even primates, including timing and regulatory mechanisms (Table 1) [30,63,64]. However, most commercial media are Mouse Embryo Assay (MEA) certified, which does not guarantee compatibility with the human embryo [65]. The composition of commercial embryo culture media remains variable [66]. Studies show that none of the commercially available formulations for culturing human embryos have analogs. Even media from one manufacturer released under different brands differ significantly in constituents, e.g., lactate, glycine, and potassium. Moreover, the components may be unstable during both cultivation and storage [66]. At the same time, the production of commercial media is often accompanied by a lack of full disclosure of the composition, and many components do not have sufficient scientific justification, which reduces confidence in their safety and the possibility of optimization [66,67]. This complicates the standardization and reproducibility and raises serious concerns about the possibility of epigenetic rearrangements, the formation of long-term developmental and health disorders in the offspring [65,68]. Studies show that different commercial media can cause imprinting disruptions indicating epigenetic instability [69]. This is supported by studies with sequential gene expression analysis of embryos cultured in different commercial media [70]. For example, significant differences in the activity of key genes at early stages of development, when cultured through to the morula stage (day 2), were observed in Ferticult and Global commercial media. Two hundred sixty-six differentially expressed genes were identified, of which 19 have a critical role in early development. At the point of transition to the blastocyst stage, the differences were less pronounced, indicating the adaptive capacity of the embryos. However, even after short-term nutrient deficiency, embryos showed atypical changes in key genes associated with embryonic transitions. This highlights the importance of controlling the components of the culture medium, since culturing under deficient conditions can lead to long-term consequences [70]. A key practical implication follows from this: complete transparency of the composition of the commercial media, including the exact concentrations of all components, is needed to enable reasonable improvement of the culture medium and make IVF more efficient [66,67]. Transcriptome studies show that the effects of the culture environment are challenging to detect without transcriptome analysis on single embryos because standard assessments of morphology and biochemistry may miss subtle molecular differences in early development [70].

Studies on embryo culturing in various environments show that group culturing of embryos has a positive effect on embryo development and survival [84,85]. However, the quality of culture conditions cannot be fully evaluated without using a large number of human embryos and invasive methods, and even then, unsuccessful pregnancies in IVF cannot be attributed solely to *in vitro* embryo culture.

A significant limitation also lies in the subjectivity of assessing embryo quality before their transfer into the uterus. Traditional criteria are primarily based on morphological characteristics determined by the clinician, which leads to significant variability in results. The accuracy of predicting successful implantation based on morphological characteristics is only 43.9–55.3%, depending on the experience and background of the embryologist [86,87].

The introduction of time-lapse imaging (TLI) technology has enabled recording of the entire embryo development process from fertilization to transfer, including morphokinetic parameters, thereby increasing the objectivity of the assessment. An analysis of 470 transferred embryos using TLI, of which 91 resulted in live birth, showed the potential of the method to improve the prediction of pregnancy outcome [88]. The integration of artificial intelligence (AI) with deep learning algorithms based on morphological and morphokinetic characteristics has demonstrated a significant improvement in classification accuracy. In a multipolar study that included 8886 IVF embryos, AI not only reached the level of accuracy of experienced embryologists but also surpassed it by 30.8% [86,89]. Although AI can improve the accuracy of embryo selection for transfer, its application is limited to the analysis of already formed embryos. To deeply understand and optimize the processes of viable embryo formation, experimental models that reproduce the key stages of human embryogenesis are needed. Such models allow for bypassing the ethical restrictions inherent in *in vivo* embryo research and create a platform for studying early stages of development without risk for embryonic material.

Preimplantation genetic testing (PGT) methods are used to detect chromosomal aneuploidies (preimplantation genetic testing for aneuploidy, PGT-A) and other genetic disorders, especially of monogenic origin (PGT-M). Polar bodies, blastomeres, and trophectoderm cells from IVF embryos can be used for PGT analysis [90]. Polar body analysis provides information mainly on the maternal genes, without a comprehensive genetic evaluation of the embryo. Blastomere analysis involves the use of 1–2 blastomeres, but this amount of genetic material is not always sufficient for an accurate assessment. The most accessible in terms of the amount of genetic material is the analysis of trophectoderm cells. A less invasive analysis is performed on trophectoderm cells at the E5-6 blastocyst stage, which subsequently do not form embryonic tissue [90]. However, the first stage of PGT is based on subjective assessment in selecting embryos with the correct morphological characteristics. On the other hand, since chromosomal mosaicism in preimplantation embryos and aneuploidy in trophectoderm cells are common, PGT-A of trophectoderm biopsy comprising only 5–10 cells does not demonstrate representativeness of the whole embryo karyotype and cannot be predictive in embryo transfer and its developmental outcome [91,92,93]. In the clinical study, seventy-seven frozen-thawed mosaic and aneuploid blastocysts were transferred to thirty-two women, resulting in nine clinical pregnancies, from which five karyotypically normal babies were delivered. This questions PGT-A comprehension and underlines rescue mechanisms for aneuploidy of an embryo [94]. The mosaic embryo development was modeled in gastruloids, demonstrating that self-organization, lineage formation, and normal spatial patterning were rescued by cells with a normal karyotype, while aneuploidy was kept only in extra-embryonic tissue [94]. The model investigation showed the mechanism of a putative rescue by cells with normal karyotype and elimination of cells with an abnormal karyotype through apoptosis during lineage specification, while aneuploid cells were allocated to extra-embryonic layers. At the same time, since accumulating data indicate misclassification of meiotic aneuploidies as mosaic, PGT-A does not seem to be absolutely accurate and requires improvement [95,96,97]. Thus, visual assessment methods and PGT-A are based on many years of practice and IVF statistics, but the success rate of the procedure is only 30% [98]. Therefore, the development of objective criteria for assessing the quality of healthy blastocysts that will develop *in vivo* and lead to successful birth, as well as simulating the processes of preimplantation embryogenesis and reaching the primitive streak stage, are complex tasks, especially *in vitro* [13,99,100,101].

**Figure 2:**
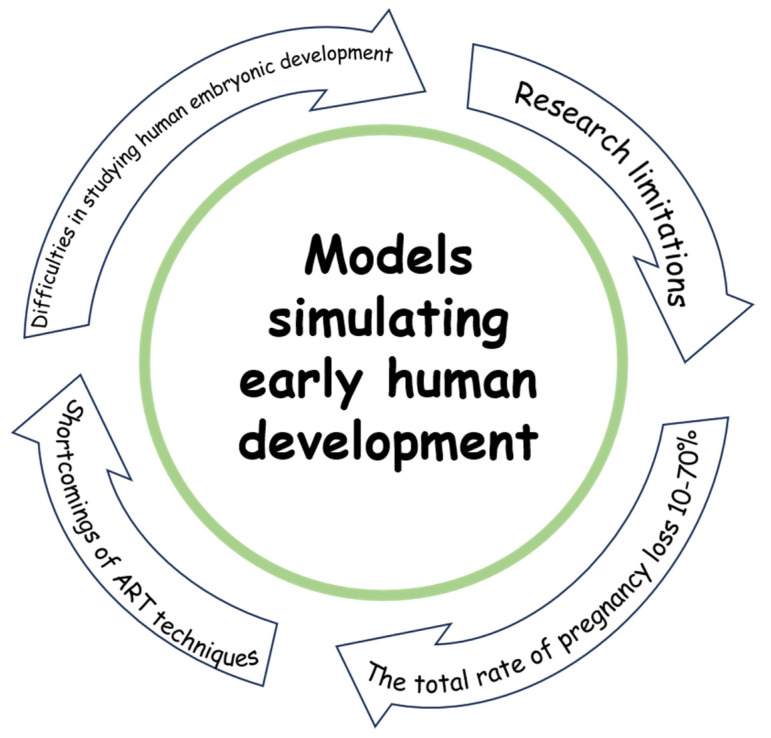
Rationales for creation of a model of early human embryogenesis.

Understanding the mechanisms of early human embryo development *in vitro* is a pressing issue that requires the creation of a convenient and reproducible model of human pregastrulation embryogenesis (Figure 2). Such models obtained *in vitro* from PSCs should have reference characteristics and imitate the human embryo. Such SES can serve as a successful alternative for fundamental studies of the processes of early human embryo development without the use of human embryos [4,102].

## 3. Blastocyst-Like Structures

During development, the embryo undergoes morphogenetic transformations that lead to the loss of totipotency by the blastomeres and the acquisition of the pluripotency status by the cells of the ICM of the blastocyst [103,104,105]. Morphokinetic and morphogenetic processes lead to the formation of a layer of epiblast cells in the peri-implantation blastocyst, which mature and, as the blastocyst undergoes implantation, pass from the rosette pluripotent state to the pregastrulation primed pluripotent state [103,106,107,108,109]. Thus, they obtain the potential to differentiate into three germ layers [110,111]. Mouse embryonic stem cells (mESCs) can be isolated from blastocyst ICM, which exhibit a naïve pluripotency state *in vitro*, while human blastocyst-derived embryonic stem cells exhibit a primed pluripotency state similar to mouse pregastrulation epiblast cells [9,10,112,113,114,115,116,117,118]. At the same time, human induced pluripotent stem cells (hiPSCs) obtained by reprogramming skin fibroblasts with pluripotency factors OCT3/4, SOX2, KLF4, and cMYC (OKSM) are capable of self-renewal and differentiating into three germ layers, giving rise to all major cell types of the body [11,12,119,120].

Ethical, diagnostic, and technical limitations due to the lack of data on early human embryogenesis led to the development of a model utilizing PSCs to simulate the blastocyst. Previously, only totipotent stem cells were thought to be capable of giving rise to both embryonic and extraembryonic cells. However, recent studies confirm the ability of human PSCs (ESCs and hiPSCs) to differentiate into extraembryonic lineages. Thus, Tietze et al. showed that upon activation of the EGF and WNT pathways and suppression of TGF-β, hPSCs form TSCs [121]. In turn, Yang et al. demonstrated that naïve hPSCs are able to differentiate into trophectoderm-like cells first and then into TSCs. The key regulator of this process is the co-factor VGLL1 (vestigial-like family member 1) acting in concert with TEAD4, GATA3, and TFAP2C, which leads to chromatin opening (H3K27ac) and activation of trophoblast-specific genes [122]. In addition, Okubo et al. demonstrated that naïve hPSCs can differentiate into hypoblast-like cells upon activation of FGF and BMP4 under conditions of TGF-β inhibition [123]. mESCs exhibit limited potential and differentiate predominantly into germ layer cells and primitive endoderm (PrE) [124,125,126]. Induction of naïve mESCs by growth factors Wnt3a and Activin A in the absence of insulin leads to the formation of PrE cells expressing *Pdgfra* and *Gata6*. Following injection into mouse embryos at E4.5, they integrate into the primitive endoderm and participate in the development of both parietal and visceral endoderm [127]. In contrast, mouse TSCs, characterized by the expression of *Eomes*, *Esrrb*, *Cdx2*, and *Tfap2c*, can be generated from mESCs through targeted genetic modifications [128]. Their formation is achieved either by overexpression of the *Gata3* and *Cdx2* regulators or by reducing the expression level of *Oct3/4* [129,130,131].

Various reprogramming protocols are known to reprogram primed hPSCs into a naïve pluripotent state [29,132,133]. In these protocols, a combination of inhibitors and inducers activates self-renewal pathways, maintains pluripotency, and/or inhibits mechanisms responsible for differentiation into the three germ layers [28,104,134,135,136,137,138,139]. However, these characteristics are not sufficient to reproduce the blastocyst-like structure, since the natural human blastocyst also includes extraembryonic cell populations (trophectoderm and primitive endoderm) in addition to cells of the embryo itself [140]. Therefore, the creation of blastoids requires conditions that ensure not only the differentiation of PSCs, but also the formation of extraembryonic compartments, which is key to imitating early embryonic development *in vitro*.

The first attempts to generate blastocyst-like structures were made using mESCs, which under certain conditions showed the ability to self-organize and form structures resembling blastocysts in morphology and organization of cell types. These structures were referred to as blastoids [16]. Later, mouse blastoids were generated using extended pluripotent stem (EPS) cells derived either from mESCs or by reprogramming fibroblasts [141,142]. Mouse blastoids demonstrated the ability to implant and induced decidualization but did not support full-fledged embryonic development. Their morphogenesis at stages corresponding to the mouse embryo E6.5–E8.5 was accompanied by arrested development and morphological abnormalities [143].

The creation of blastocyst-like structures in mice served as the basis for the development of similar protocols in humans [16,141,142]. Using the differentiation potential of human PSCs, it became possible to form three-dimensional structures that imitate the morphology and cellular organization of the blastocyst and are now considered promising model systems for studying the processes of implantation and early differentiation in humans [17,18,19,20,21,22,143,144].

## 4. Generation of Human Blastoid

**Figure 3:**
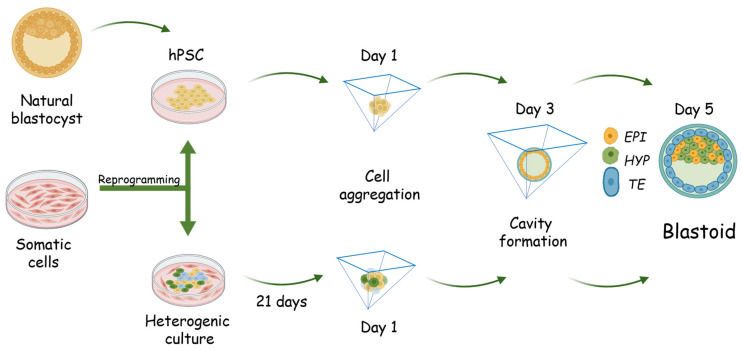
General scheme of blastoid generation. The figures were made using the online editor BioRender.

Researchers have used three main strategies to generate human blastocyst-like structures. The first strategy is based on the ability of naïve hPSCs to differentiate into extraembryonic cell types such as trophoblast and hypoblast cells, which leads to the correct topological and morphological self-organization of blastocyst-like structures (Figure 3) [19,20,21]. The second strategy is to use EPSCs, which are also capable of differentiating into extraembryonic cells [15,145]. Such cells are a convenient tool for generating chimeras and facilitate the development of synthetic embryology [15,146,147]. Inhibition of SRC, GSK-3β, and Tankyros leads to the formation of human EPSCs that upregulate genes such as *DPPA3* (*Stella*), *ZSCAN4*, *KLF4*, *TFAP2C* and classical pluripotency factors *OCT3/4*, *SOX2*, and *NANOG* and reduce global DNA methylation levels compared to naïve and primed EPSCs [15,145]. This contributes to the maintenance of a more flexible and extended state of pluripotency characteristic of early embryogenesis. At the same time, EPSCs experience a decrease in global DNA methylation reflecting their epigenetic plasticity and chromatin openness. Such hypomethylation creates favorable conditions for the activation of genes required for totipotency and expands the differentiation potential of EPSCs into both embryonic and extraembryonic lineages [15,145,147]. The main difference between these cells and naïve PSCs is their histone gene expression, which is comparable to that of blastomeres from eight-cell embryos. When generating human blastoid cells, EPSCs are first differentiated into TSCs and then both cell types are co-cultured in 3D conditions for self-organization [17,142]. Another strategy is to use a standard protocol for reprogramming human fibroblasts into iPSCs with OKSM essential pluripotency factors, which enables the formation of all cell types of the preimplantation embryo [18]. Then, trophoblast-, hypoblast-, and epiblast-like cells are forced to self-organize to form a blastocyst-like structure with a cavity and a cluster of compact cells similar to the blastocyst ICM.

To generate blastoids with cavity, the aggregate size was strictly controlled by the number of cells per aggregate. While mouse blastoid formation started with five cells per aggregate, the initial cell content for human blastoid development was 50–100 cells per aggregate [16,17,18,20,22].

**Figure 4:**
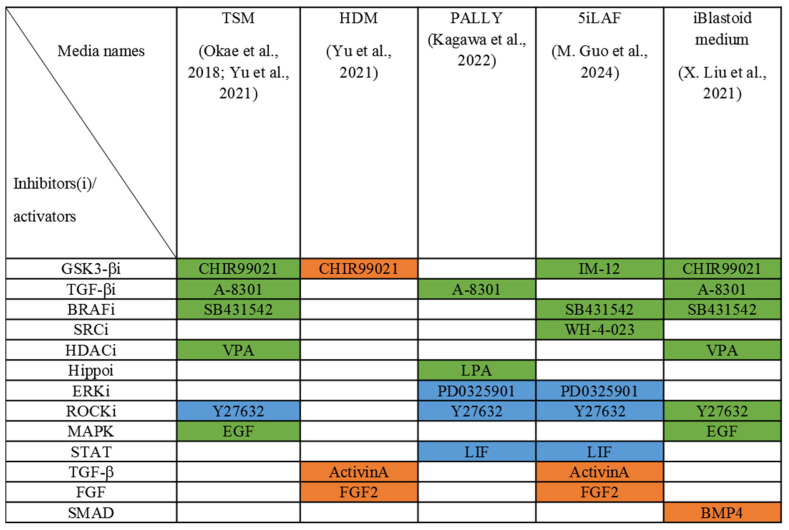
Factors influencing the differentiation of the main cell types of the human blastocyst into blastoids. Green—trophoblast-stimulating factors; orange—hypoblast or primitive endoderm; blue—supportive factors [18,19,20,21,148].

After the formation of cell aggregates from hPSCs, the subsequent formation of blastocyst-like structure was controlled by GSK-3b (CHIR99021, IM-12), transforming growth factor β (TGF-β) (A-8301, SB 431542), and ERK (PD0325901) pathway inhibitors to direct the differentiation of naïve hPSCs towards trophectodermal and hypoblast-like cells [17,18,22,144]. ROCKi (Y27632) was used not only to enhance cell survival but also to maintain the integrity of blastocyst-like structures (Figure 4).

In the work of Yu et al., to obtain blastoids, naïve hPSC aggregates were treated first with a medium for differentiation into the hypoblast and then with a medium for differentiation into the trophectoderm [19]. This sequence, although not canonical, showed the best efficiency. The authors emphasized that the components of the differentiation medium for the direction of naïve hPSC aggregates into trophectoderm were inhibitors of the TGF-β and ERK pathways, while activation of TGF-β -SMAD and WNT-β-catenin signaling together with fibroblast growth factor (FGF) is necessary for hypoblast induction. Using the protocol of Yu et al., another research group showed that cavitation in human blastoids was associated with activation of the AKT/mTOR pathway and MAPK3/1 [149]. Moreover, they noticed higher ratios of phosphorylated forms of AKT, MAPK3/1, and RPS6KB1 in blastoids versus non-blastoid aggregates. Additional studies also reported activation of PI3K/AKT and mTOR pathways during blastoid formation, supporting their significance in morphogenesis [23]. In contrast, Kagawa et al. used the Hippo pathway inhibitor lysophosphatidic acid (LPA) together with inhibition of the ERK and TGF-β pathways [20], which had a positive effect on differentiation towards trophectoderm [150]. The early specification of trophectoderm-like cells into a blastocyst-like structure is determined by the nuclear translocation of YAP1 and its binding to the transcription factor TEAD4, which activates the expression of trophectoderm markers including DAB2, CDX2, GATA2, and GATA3 [20]. Thus, blastoid trophectoderm morphogenesis is determined by the nuclear localization of YAP1 and the positive expression of CDX2, which is further supported by the active expression of *GATA3* and the activation of protein kinase C (aPKC), which involvement in lumen formation was demonstrated before. In turn, phospholipase C (PLC) responsible for the polarization and further epithelialization of peripheral cells in aggregates induces cavitation in the blastocyst-like structure [20,151,152,153]. Maintenance of a naive ICM-like state is mediated by activation of the LIF/STAT3 pathways and suppression of TGF-β/Nodal and ERK signaling [22]. The addition of LIF enhances blastoid formation and influences epiblast/trophectoderm interactions through GP130/STAT3-dependent mechanisms [20]. The specification of the hypoblast in human blastoid is regulated by the FGF/MEK/ERK signaling, which is required for the activation of the transcription factors GATA4 and GATA6 [154]. Additionally, the BMP/SMAD pathways may interact with the FGF/ERK cascade modulating the balance between the epiblast and hypoblast lineages, which is consistent with observations in early embryogenesis [155]. RNA-seq analysis of human blastoids revealed the upregulation of WNT3 and RSPO3, confirming the involvement of WNT/Nodal signaling in the formation of hypoblast-like cells [20]. In this work, a natural sequence of blastoid developmental processes is observed, demonstrating the greatest morphological and phenotypic similarity of the blastoid to the human blastocyst.

The work of Guo et al. differs from the above protocols, as it did not use specific media for differentiation into the main cell types of the blastocyst [21]. In this work, blastoids were obtained under culture conditions for naïve PSCs (called 5iLAF) by inhibiting five signaling pathways: MEK, GSK3, BRAF, ROCK, SRC, activation of STAT3 by LIF, and induction of ActivinA (TGF- β) and FGF2 [21,138]. The formation of these so-called spontaneous blastoids under 5iLAF conditions can be explained by several factors. According to the results of transcriptional analysis, there is a division into subpopulations of hypoblast cells and GATA3-positive trophoblast cells with different degrees of preimplantation maturity in a naïve hPSC culture under these conditions [156]. Comparison of different blastoid culture conditions (Figure 4) shows that almost all components required for trophectoderm and hypoblast specification are present in 5iLAF, and due to the initial heterogeneity of the naïve hPSC population, the formation of blastocyst-like structures is actively initiated [21,156]. A distinctive feature of this method is the use of IM-12 as a GSK-3β inhibitor. According to the authors, this inhibitor influences the ability of naïve hPSCs to self-organize into blastocyst-like structures and allows for the efficient production of blastoids [21]. The authors analyzed molecular differences between cells generated with IM-12 and another GSK-3β inhibitor, CHIR99021, showing that IM-12 modulates the activation of signaling pathways that lead to increased expression of genes associated with oxidative phosphorylation (*OXPHOS*), extracellular matrix (*LAMB3*, *COL3A1*), and cell adhesion genes (*PCDH7*). These genes, in turn, affect the efficiency of blastoid formation [21]. By inhibiting GSK-3β, CHIR99021 activates the canonical WNT signaling pathway and prevents the formation of 3D structure but preserves naïve pluripotency of cells in 2D culture. At the same time, naïve hPSCs obtained in the presence of CHIR99021 but subsequently transferred to a medium with IM-12 do not form blastoids, which demonstrates the importance of using IM-12 at the stage of reprogramming primed hPSCs to a naïve state and then in obtaining and culturing blastoids [21].

In addition to naïve hPSCs, EPSCs were used to form blastoids [17]. In the study by Fan et al., the researchers used the data from Sozen et al., who had shown the ability of mouse EPSCs to form blastoids when exposed to an induction medium containing BMP4 [142]. To modify the protocol of Sozen et al., EPSCs were pre-induced into trophectoderm-like cells with BMP4, then mixed with uninduced EPSCs, and allowed to self-organize in round-bottom plates. Despite the inclusion of induced trophectoderm-like cells, the efficiency of blastoid formation remained low, highlighting the challenges of maintaining stable, extended pluripotency in human EPSCs [17]. EPSC maintenance remains technically challenging, as the stability and reproducibility of the EPSC state vary, necessitating additional research [17,145].

Let us analyze a strategy for generating blastoid cells obtained not from naïve human hPSCs, but through the reprogramming of somatic cells into hiPSCs (Figure 3) [18]. This protocol is unique because it enables the generation of blastocyst-like structures directly from human skin fibroblasts, bypassing the stages of transition to a naïve/expanded state, as well as targeted induction into blastocyst cell analogs. As mentioned above, under standard conditions, after reprogramming somatic cells into hPSCs, the latter have the status of primed pluripotency [11,157,158]. Currently, a protocol exists for differentiating primed hPSCs into trophoblast cells [121]. However, the protocol of Liu et al. demonstrates the potential to obtain all major blastocyst cell types under standard reprogramming conditions. In iPSCs reprogrammed from somatic cells, regulatory elements and promoters of pluripotency-associated genes are activated due to chromatin reorganization and accessibility, while the accessibility of somatic regulatory elements is reduced [159]. Epigenetic reorganization in the cell being reprogrammed is consistent with a bifurcation of the reprogramming trajectory through primed or naïve pathways and depends on the conditions applied to achieve different pluripotency states [159]. In the absence of naïve or primed pluripotency conditions, reprogramming somatic cells follow different reprogramming pathways. They can acquire a naïve pluripotency state without going through the primed state [159]. In addition, it has been shown that the presence of OKSM reprogramming factors is sufficient to obtain subpopulations of trophoblast, hypoblast, and epiblast cells simultaneously, even under fibroblast culture conditions [159]. The formation of such a heterogeneous cell population during reprogramming of somatic cells into iPSCs, trophoblast, and hypoblast is possible when the silent heterochromatin of somatic cells turns into euchromatin at the initial stages of reprogramming. This happens due to the balance of the main transcription factors of pluripotency and the gradient of their binding to the regulatory elements of pluripotency genes [160,161,162]. Heterogeneous cultures of primed hPSCs can include subpopulations of naïve hPSCs and/or totipotent stem cells, which are capable of differentiating into both germline and extraembryonic cells [10,148,158,163,164,165]. The primate epiblast is considered to have greater plasticity than the mouse epiblast. Studies also confirm that primed hPSCs exhibit increased plasticity under certain conditions *in vitro* and during embryonic development [166].

Liu et al. showed that by day 21, three main types of blastocyst cells appear in the culture of reprogrammed fibroblasts, so it was decided to continue culturing under non-adhesive conditions to obtain cell aggregates consisting of 100–300 cells of the resulting heterogeneous mixture [18]. This led to the formation of 3D cell aggregates and their further cavitation, and by day 6 of culturing under new conditions, iBlastoids were formed. It is worth noting that the blastoid culture medium enriched with hormones (β-estradiol and progesterone), bovine serum, and containing inhibitors (A83-01, VPA) promotes the development of the trophectoderm analog (Figure 4), allowing cell aggregates to cavitate and form blastoids [18].

Thus, the formation of human blastoids represents a finely balanced integration of multiple signaling cascades, including Hippo, WNT/Nodal, FGF/ERK, BMP/SMAD, LIF/STAT3, and AKT/mTOR pathways, which act in concert to coordinate self-organization, spatial segregation, and lineage stabilization. The cross-regulation among these pathways governs key transitions from the pluripotent state toward lineage specification of trophectoderm, epiblast, and hypoblast, as well as the morphogenetic processes leading to cavity formation. Perturbation of this signaling balance results in loss of symmetry and disruption of blastoid architecture, underscoring the essential role of coordinated signaling in early human embryogenesis. For a more comprehensive overview of the signaling mechanisms underlying blastoid formation, one can refer to the reviews by Jiao et al. and Moinard et al. [167,168].

## 5. Characterization of the Forming Blastoids

**Figure 5:**
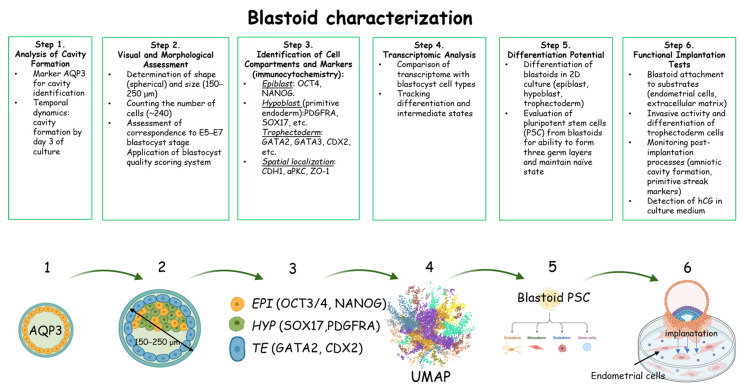
Step-by-step scheme of blastoid characterization for tracking the formation of a blastoid with characteristics most consistent with a natural human blastocyst. The figures were made using the online editor BioRender.

As mentioned above, the blastoid is an imitation of the human blastocyst, so it should correspond to its characteristics. The main steps of blastoid characterization are summarized in Figure 5. After induction of the cell aggregates into blastoids, the AQP3 marker was used to track cavity formation in them. Initially, it was detected in all blastoid cells, and subsequently it remained only in trophectoderm cells [20]. Blastoid cavitation usually occurred on the 3rd day of cultivation [17,18,19,20]. While blastoids were forming, the blastocyst quality assessment scale for IVF was used for the morphological and topological assessment of their quality [18,19]. It allows for visual assessment of the ICM and trophectoderm. To assess compartment specific blastoid cell contacts, the adherens junction marker CDH1, the apicobasal polarity marker aPKC, and the tight junction marker ZO-1 were detected. To determine the blastoid growth rate, the proliferation markers EdU and Ki67 were used [18,19]. The spatial topology of these cell types in the formed blastoids was determined using the immuno-cytochemistry method. Thus, on the surface and along the periphery of the blastoid, there were analogs of trophoblast cells (GATA2, GATA3, CDX2), an analog of the epiblast (SOX2, OCT4, NANOG) adhered to the polar trophectoderm (CCR7, NR2F2) inside, and an analog of the hypoblast (SOX17, GATA6) laid on the epiblast surface. When characterizing blastoids after determination of the spatial topology of the cell types, three main compartments were distinguished, which are analogs of the epiblast, hypoblast, and trophectoderm of the blastocyst, and expressed the corresponding markers. The blastoid epiblast analog was confirmed by the expression of major pluripotency marker genes, such as *SOX2*, *OCT3/4*, *NANOG*, and naïve pluripotency marker genes *OTX2*, *CD24*, *KLF4*, *KLF17*, *SUSD2*, *DPPA2*, *ATG2A*, *TFCP2L1*, *PRDM14*. The hypoblast analog, or primitive endoderm, was defined mainly using GATA6 and SOX17 markers. To determine mature hypoblast cells, a combination of markers, such as PDGFRA, SOX17, GATA4, GATA6, was used [17,18,19,20]. The trophectoderm analog expressed such key markers as GATA2, GATA3, CDX2, DAB2 [17,18,19,20]. At the same time, to track further development and events that mimic implantation, the authors identified expression of polar trophectoderm genes *CCR7*, *MUC15*, *OVOL1*, *CYP19A1*, and *NR2F2* [17,18,19,20].

Using the protocols described in the previous paragraphs, parameters of the formed blastoids, such as the correct spherical shape and proportional combination of cellular compartments, were shown to correspond to those of the human blastocyst at the E5–E7 stages [17,18,19,20]. The size (150–250 μm) and the total number of cells (about 240) were also comparable.

Another method to confirm the similarity between a blastoid and a blastocyst is transcriptome analysis. The three major cell types of blastoids showed high transcriptional similarity with the corresponding epiblast, hypoblast, and trophectoderm clusters of blastocysts, while blastoids were transcriptionally distinct from postimplantation embryos [17,18,19,20]. Blastoids had the correct cell lineage directions, which were studied by RNA velocity analysis to assess transcriptome changes over time [19,20,21]. This method allows tracking the origin and specification of blastoid cell types at certain points in time and identifying intermediate clusters of cells that respond to culture conditions that cause cell type specification.

Primed hPSCs and trophectoderm stem cells, which will subsequently differentiate into embryonic and extraembryonic tissues, can be obtained from the blastocysts. Therefore, blastoid cells were subjected to differentiation potential studies [10,162]. For this purpose, analogs of epiblast, hypoblast, and trophectoderm cells were obtained from blastoids and differentiated in 2D culture [17,18,19,20]. PSCs obtained from the blastoid ICM compartment were able to differentiate into the three germ layers. When naïve pluripotency conditions were applied, blastoid PSCs maintained a naïve state consistent with PSC characteristics [17,18,19,20]. Trophectoderm cells demonstrated the ability to develop into syncytotrophoblast (SDC1^+^) and extravillous cytotrophoblast (HLA-G^+^) while losing the trophectoderm stemness marker (CHD1) and expressing cytotrophoblast genes (*GATA3*, *CK7*, *CGB*). Hypoblast cell counterparts differentiated into extraembryonic lineages, such as extraembryonic mesenchyme (COL6A1^+^), visceral endoderm, and yolk sac (FOXA1^+^) [17,18,19,20]. Another method for assessing differentiation potential is the ability to form chimeras. In the work of Yu et al., naïve PSCs from a blastoid were microinjected into a mouse blastocyst followed by *in vitro* culturing. The percentage of the contribution of injected cells to each host cell compartment was determined after 5–6 days of culture. Trophoblast cell derivatives (GATA3^+^) accounted for 23.9%, hypoblast cells (GATA6^+^)—for 8.0%, and naïve ESCs (SOX2^+^)—for 15.8% of all mouse embryo cells [19]. This method is the gold standard for characterizing naïve PSCs [169].

Blastoid functional evaluation was performed using an *in vitro* blastoid implantation assay. For this purpose, blastoids were seeded on various substrates, such as endometrial cells or basement membrane-mimicking scaffolds, using embryo culture media, e.g., IVC1/2 [17,18,19,20]. Within the first 24 h, blastoids attached to the substrate, and trophectoderm cells invaded the substrate differentiating into extravillous trophoblast and syncytotrophoblast, which formed multinucleated cells [17,18,19,20]. At the same time, a clear division of the ICM compartment into epiblast and hypoblast cells was evident. SOX2- and OCT4-positive cells characterizing the epiblast were observed in the center of the attached blastoids, while GATA6-positive cells characterizing the hypoblast were observed around them. Blastoid culturing was continued after its attachment for several more days to track the main events of implantation: postimplantation epithelialization, acquisition of the primed state by epiblast cells (positive for markers F-actin, CD24, DPPA2, GDF3), formation of the pro-amniotic cavity (positive for markers F-actin, PODXL, aPKC), definitive endoderm, and yolk sac. To control the onset of gastrulation and avoid ethical restrictions, the authors checked for the presence of primitive streak markers (*TBXT*, *EOMES*) in attached blastoids. To assess the quality of blastoid implantation and the functioning of extravillous trophoblast cells, the conditioned medium from the blastoids was collected and tested for human chorionic gonadotropin (hCG), yielding a positive result [18,19,20,21]. Thus, functional tests demonstrated that blastoids are capable of imitating the implantation and pregastrulation processes of the blastocyst.

## 6. Application of Blastoids

One of the key areas of application of blastoids is the study of implantation. Blastoids are a powerful tool for studying early implantation and embryo-endometrium interactions [19,20,22,24]. Modern models combining blastoids and hormonally activated endometrial organoids reproduce the key stages of implantation (apposition, adhesion, and invasion), as well as the interaction of trophoblast with endometrial cells [23,170]. This makes it possible to study the molecular mechanisms of successful implantation and the causes of its impairment, which is of practical importance for ART. In particular, blastoids can be used to develop and test methods to improve embryo implantation, assess the functional state of the endometrium, and analyze the effects of the drugs on early pregnancy without the need to use natural embryos.

Blastoids provide a platform for systemic analysis of epigenetic patterns and transcriptional trajectories that are inaccessible when working with natural embryos. Recent studies have shown that blastoid culture facilitates the modeling of early stages of gastrulation, the formation of embryonic axes and tissues, thereby opening new opportunities to investigate gene regulation and the influence of environmental factors on early development [24]. These models can be used to investigate the causes of early embryonic mortality, implantation disorders, and hereditary diseases, as well as to assess the safety of drugs in early embryogenesis.

Protocols have been developed to obtain blastoids with high efficiency (>70%) using Hippo, TGF-β, and ERK inhibitors, ensuring reproducibility of experiments [20,23]. In the future, blastoids can be cryopreserved to create biobanks for large-scale studies, including drug screening and toxicology testing, without the use of human embryos.

In addition to humans, blastoids have been successfully obtained from other species. For example, in cattle, blastoids generated from EPS and TSC exhibit similarities to natural blastocysts in morphology, cell lineage composition, and functional activity, including induction of maternal interferon-τ (an analog of hCG) after intrauterine transfer [171]. The creation of a cynomolgus macaque embryo model using blastoids generated from naïve PCSs through a two-step protocol has also been reported [19,172]. The resulting model successfully recapitulates key stages of embryogenesis, e.g., gastrulation and early implantation, and may serve as a valuable tool for studying the mechanisms of early embryonic development and embryo-maternal interactions. Blastoids from the cynomolgus macaque demonstrate invasive behavior towards the endometrium allowing to model early pregnancy *in vivo*. This opens up new possibilities for studying the causes of implantation failures and early embryonic mortality, as well as for developing therapeutic strategies in the field of reproductive medicine [172]. Together, these models open up new directions in animal husbandry, reproductive biology, and the study of interspecies differences in early embryogenesis [171,172].

Current approaches may enable the creation of personalized blastoids from iPSCs obtained from specific patients, which gives the possibility of modeling individual features of implantation and early development. The generation of patient-specific blastoids based on hiPSCs is considered a proof-of-concept for modeling personalized aspects of implantation, early embryonic development, and reproductive pathologies. This approach was first demonstrated in studies using human iPSCs to generate blastoids, followed by comparative analyses of their morphology, transcriptomic profiles, and capacity to attach to endometrial organoids [19,20,22,23]. The study showed that blastoids can recapitulate key signaling interactions, such as the Hippo, WNT, FGF, and TGF-β pathways, characteristic of early embryogenesis, confirming the feasibility of individualized reconstruction of early developmental stages [18,19,20,22,23].

Subsequently, Heidari Khoei et al. developed a reproducible protocol for co-culturing human blastoids with endometrial organoids, providing proof-of-concept evidence for functional implantation testing *in vitro* [20,22]. This approach lays the groundwork for personalized and autologous models, in which both embryonic and endometrial cells originate from the same donor, enabling the study of patient-specific implantation dynamics. Moreover, studies by Yu et al. and Fan et al. demonstrated that the use of iPSCs derived from different donors leads to observable differences in blastoid morphogenesis and gene expression patterns, supporting the concept of genetic and epigenetic individuality in early developmental models [17,19,23]. These individual variations can be leveraged to predict implantation potential and pharmacological responses, opening perspectives for precision reproductive medicine.

Thus, personalized blastoids represent a proof-of-concept system that integrates individual genetic information with physiological modeling of key developmental processes, such as implantation, trophoblast invasion, and embryo-endometrial signaling. They pave the way for the development of *in vitro* platforms for personalized reproductive testing, drug screening, and predictive diagnostics of infertility while minimizing ethical concerns [173]. Personalized blastoid models represent a promising direction for future research in ART and human developmental biology.

**Figure 6:**
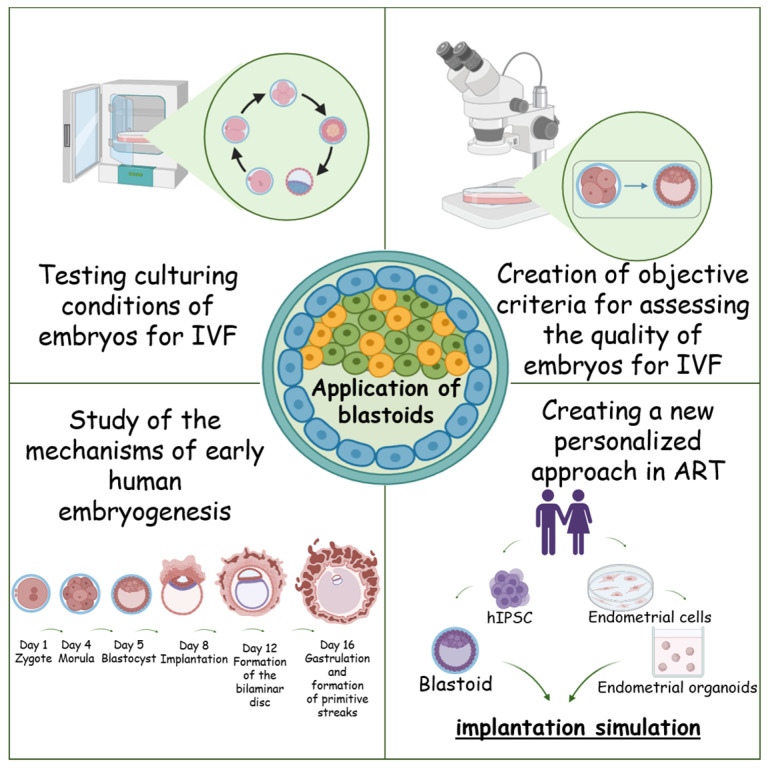
Potential of using blastoids in ART and for studying early human embryogenesis. The figures were made using the online editor BioRender.

Additionally, blastoids can serve as a model for studying intercellular communication via exosomes and extracellular signaling molecules, which allows us to understand the early mechanisms of regulation of embryonic development and identify new biomarkers for successful implantation. Thus, blastoids represent a multifunctional and scalable model that integrates the study of early embryogenesis, implantation, epigenetic regulation, and interspecies differences while also serving as a platform for drug screening, toxicology assessment, and fundamental research in regenerative medicine (Figure 6). Their application in ART and personalized approaches allows the development of new therapeutic strategies to reduce the risks of early embryonic mortality and implantation failure, as well as to study individual features of embryonic-endometrial interactions that are inaccessible when working with natural embryos (Figure 6).

## 7. The Problem of Identification of the Hypoblast-Like Compartment of the Blastoid

The above methods of blastoid characterization are the main ways to determine whether a blastocyst-like structure corresponds to a human blastocyst. Since the studies do not always present all morphological, morphogenetic, and functional tests, the question of creating a single list of criteria remains open. These criteria can become a standard for blastoid characterization, similar to those used to characterize new PSC lines. In our opinion, functional tests for basic characteristics, such as morphology, size, phenotyping of the main cell types of blastoids, and transcriptome analysis, should be carried out in a complex. Only after confirming the correspondence of these parameters to the blastocyst parameters, the structure can be designated as a blastoid. Otherwise, utilizing structures that do not correspond to the parameters of blastoids can give incorrect results.

None of the above methods allows for obtaining a complete analog of the human blastocyst. All models have certain disadvantages. The main problem of almost all models is the absence of a visible hypoblast cluster or its incompleteness [17,18,21]. The low efficiency of obtaining hypoblast lines in human blastoids may be due to the fact that the hypoblast in a natural blastocyst is formed later than the epiblast and trophectoderm cells, and hypoblast markers are often expressed in trophectoderm cells. In mice by E4.5, a clear division into two phenotypically and topologically different layers, the outer layer GATA6^+^SOX17^+^ (PrE) and the inner layer NANOG^+^SOX2^+^ (epiblast), occurs [174,175]. In humans, however, only 6 days after fertilization does the blastocyst show a transcriptome profile separation between epiblast cells that are OCT4^+^/NANOG^+^/GATA6^−^ and hypoblast cells that are OCT4^+^/GATA6^+^/NANOG^−^ [176]. Moreover, these cell types are not physically separated in the ICM compartment of the human blastocyst [13]. On the seventh day of embryonic development, the polar cells of the trophectoderm acquire the ability to bind to the substrate, and the embryo becomes elongated. Already by the 8th day after fertilization (corresponding to the Carnegie stage 5a), spatial and topological segregation of ICM cells into the epiblast and hypoblast occurs, forming a bilaminar embryonic disc. The problem of clear visualization of the hypoblast in the blastocyst-like structure may be related to the phenotyping of hypoblast cells, since the activation of hypoblast genes takes place sequentially during the early stages of human blastocyst development [177]. The article by Corujo-Simon et al. showed that in mice, GATA6 is a universal and reliable early marker of primitive endoderm specification, whereas in humans, its expression is more heterogeneous and does not always clearly correlate with hypoblast formation [177]. However, in the above studies, GATA6 was used as the main marker of the human hypoblast, which should lead to incorrect data [17,18,19]. It is more appropriate to identify the human hypoblast at the early stages of cell specification using a combination of several markers, for example, PDGFRA in combination with SOX17, as shown by Corujo-Simon et al., while GATA6 is a common preimplantation human extraembryonic marker that is expressed in both trophoblast and hypoblast cells [177]. This should change the strategy for phenotyping epiblast at the early stages of blastocyst-like structures development and will allow more accurate tracking of the lineage formation in human blastoids.

Despite the fact that the media in all studies contain factors influencing the specification of the hypoblast (Figure 4), the hypoblast-analog compartment is not fully represented. This shows that such blastocyst-like structures do not meet all the criteria for corresponding to a blastocyst. For example, in the work of Guo et al., incomplete hypoblast segregation was also observed when culturing in 5iLAF [21]. According to the authors, this was due to the BRAF/MEK inhibitors used. Their removal initiated hypoblast segregation, but this reduced the efficiency of blastoid formation [21]. These data confirm the need to optimize blastoid cell production protocols and culture conditions, since existing culture conditions are insufficient to form analogs of the three main blastocyst cell types in a 3D structure.

Another issue related to blastoid formation is the presence of a population of cells that do not belong to blastoid cells but survive in culture and are detected by transcriptome analysis. For example, in a mixture of blastoid cells obtained by reprogramming human fibroblasts, the authors define such cells as resistant to reprogramming and not belonging to the blastocyst-like structure [18]. In addition, all studies analyze the transcriptome and detect intermediate clusters, in some cases much larger in cell number than the three main clusters corresponding to the main cell types of the blastocyst [17,18,19,20,21]. These clusters likely represent intermediate states during the specification of the main blastoid cell types, and their study may provide insight into how these cell types are formed [20].

Some models show deviations in rates and pathways of development compared to natural embryos, which requires further evaluation [21]. This phenomenon has been observed in other SES as well [178,179]. This may indicate pathological processes in blastoid development and give incorrect results when using such SES.

## 8. Naïve Pluripotency Conditions for Blastoid Formation

It is worth noting that the sizes and ratios of the main blastocyst compartments vary in many studies [17,18,19,21]. For example, in the work of Fan and colleagues, the number of blastoid cells exceeds the number of cells in the blastocyst, as seen in micrographs [17]. The ICM compartment of the blastoids is much larger compared to the blastocyst, and there may also be an increased number of apoptotic cells, which has not been investigated. This may indicate abnormal developmental processes of the blastoid and may be the reason for the low efficiency of this method [17]. In the 5iLAF protocol, the resulting spontaneous blastoids did not have a proper spherical shape even after removing inhibitors that affect hypoblast specification [21]. This may be due to the disruption of epigenetic mechanisms during the culture of naïve hPSCs in 5iLAF conditions. Naïve hPSCs obtained using 5iLAF lose stable primary imprints established during the development of primordial germ cells. Such gene sites are not demethylated during preimplantation development, which distinguishes the global DNA methylation landscape of naïve hPSCs in 5iLAF conditions from that of blastocyst ICM cells [134]. This, in turn, leads to biallelic expression of imprinted genes, which can negatively influence further development and lead to karyotypic abnormalities.

A recent study on obtaining blastoids from naïve hPSCs in 4CL conditions, followed by the use of an optimized two-step protocol, showed the highest efficiency compared to the above methods [24]. 4CL conditions maintain the naïve state of hPSCs through a combination of signaling and epigenetic mechanisms: LIF activates the JAK/STAT3 pathway, maintaining Activin/Nodal signaling and NANOG expression, which contributes to the maintenance of pluripotency, while PD0325901 and IWR-1 suppress the MAPK/ERK and WNT/β-catenin pathways, preventing spontaneous differentiation. At the same time, a complex of epigenetic modifiers is turned on that reduces DNA methylation and H3K27me3 levels (EZH2 and vitamin C), creating a more plastic chromatin landscape (HDACi) [180]. This balance of signaling and epigenetic changes allows cells to remain closer to the ICM state of the embryo, thereby maintaining naïve pluripotency. The authors developed an improved human blastoid model that not only recapitulates blastocyst morphology and cell lineages but also displays epigenetic characteristics similar to natural embryos, including DNA methylation and imprinting patterns [24]. Blastoids obtained under these conditions undergo stages equivalent to Carnegie Stage 6 upon long-term culture (approximately 14 days), forming germline axes and cell populations consistent with early gastrulation *in vivo*. 4CL medium demonstrates clear advantages over 5iLAF in multiple key parameters for culturing naïve hPSCs. Cluster analysis data showed that cells cultured in 4CL have a gene expression profile more similar to ICM and 8-cell embryos than those cultured in 5iLAF [132,180]. This allows us to consider 4CL a more effective environment for converting cells into a state closer to the natural early embryo. Analysis of CpG methylation, most notably in imprinting control regions, showed that 4CL (particularly its e4CL modification) provides a state close to the methylation pattern of the preimplantation stage ICM. Thus, unlike 5iLAF, 4CL better supports the physiological epigenetic program [180].

## 9. Limitations of Blastoid Application

Blastoids are not a complete analog of the human blastocyst; however, the large number of studies on obtaining blastoids has raised several ethical and legal issues. ISSCR addressed the issue of SES, such as blastoids and gastruloids, in 2021 and released guidelines for the use of these models of human embryogenesis [42]. According to these guidelines, SES were divided into integrated and non-integrated stem cell-based embryo models. Blastoids belong to integrated stem cell-based embryo models and consist of both embryonic and extraembryonic tissues. They can successfully reproduce the implantation process and the first steps of gastrulation. They are direct “competitors” of gastruloids, since gastruloids mimic only certain stages of embryogenesis, lack extraembryonic tissues, and thus, belong to non-integrated embryo models [42]. However, due to their close similarity to the human embryo, blastoids may also fall within the scope of the 14-day rule, which will limit the use of this model in studying early human embryogenesis [173]. For example, personalized blastoids fall under the definition of a human embryo according to Australian law, so studies using them require ethical approval [18,42]. Therefore, researchers may lose the opportunity to use blastoids as SES to model early human embryogenesis. Thus, continuous ethical evaluation, transparency, and regulatory compliance remain essential to maintaining public trust as this field advances.

## 10. Future Perspectives and Research Potential

In the foreseeable future, blastoids are expected to substantially expand both the experimental and translational potential of research in early human embryonic development and ART. Blastoids have already proven to be an ethically sustainable and scalable model that recapitulates key aspects of early embryogenesis and interacts functionally with endometrial cells, making them highly suitable for *in vitro* implantation modeling [20,22]. Recent advances have demonstrated efficient methods for large-scale generation of human blastoids, enabling high-throughput analyses of blastoid–endometrium interactions and paving the way for functional genomics approaches. In particular, CRISPR-based screening strategies can now be applied to identify genes regulating lineage specification, cell survival, and implantation potential [23,181]. The availability of CRISPR and perturbation tools previously optimized in hPSC systems makes their adaptation to blastoids both feasible and timely.

From a translational standpoint, comparative transcriptomic and epigenomic profiling between normal and pathological developmental trajectories in blastoids may help uncover novel therapeutic targets to improve implantation outcomes or prevent early pregnancy loss. Moreover, the integration of AI, multi-omics datasets, and stochastic modeling is anticipated to enable the reconstruction of complex regulatory networks underlying early human development, thereby accelerating hypothesis generation and testing [182,183].

Blastoids also hold great promise as a platform for drug screening and developmental toxicity testing, since they mimic several essential stages of early human embryogenesis. This enables evaluation of compound effects on differentiation and morphogenesis, potentially reducing reliance on natural embryos. However, while preliminary proposals and regulatory documents support this application, direct evidence of blastoid-based assays that fully replicate embryo–uterine interactions in drug testing remains limited. Regulatory and ethical frameworks are still evolving.

Personalized blastoids derived from patient-specific iPSCs or directly reprogrammed somatic cells, such as human fibroblasts, are particularly promising [18,19,22,23]. These systems can reflect individual genetic and epigenetic variability, providing an opportunity to model patient-specific implantation capacity, cellular responses to microenvironmental cues, and pharmacological sensitivity. Protocols have already been developed for co-culturing blastoids with endometrial organoids, enabling the establishment of individualized *in vitro* testing systems [22,170].

For the broader application of such systems, the development of standardized protocols, cryopreservation techniques, and blastoid/iPSC biobanks is essential to ensure reproducibility, inter-laboratory validation, and scalability. Approaches for cryostorage and protocol harmonization have already been described and are adaptable for material exchange and collaborative studies [22].

Collectively, human blastoids bridge fundamental embryology and clinical reproductive research, offering a platform that combines scalability, ethical feasibility, and personalization potential. They open the way toward a deeper understanding of implantation mechanisms, discovery of biomarkers and therapeutic targets, and the next generation of diagnostic and treatment strategies for reproductive disorders.

## 11. Conclusions

The described strategies for obtaining blastocyst-like structures demonstrate that the generation of a human blastocyst model is a complex process that requires mandatory compliance with multiple factors affecting the specification and maintenance of the three main cell types of the blastocyst. They also demonstrate the broad capabilities of hPSCs to form three-dimensional structures and imitate the main events of early human embryogenesis. However, many of the presented models require modifications to culture protocols and more precise blastoid characterization, as the culture conditions they provide are insufficient for the complete specification of all three blastocyst cell types. This may be due to the incorrect choice of markers for phenotyping the hypoblast cell analog. In addition, there is a need to establish clear criteria for evaluating the obtained blastoids, as the functional tests in each protocol differ. In this regard, it is worth paying attention to the spatial transcriptomics of blastocyst-like structures.

In some cases, transcriptome analysis shows a high degree of similarity between blastoids and blastocysts; however, such structures may not meet the main criteria for cell phenotyping and the morphological features of a blastoid. Addressing these issues may enable using blastoids as human blastocyst models to study early embryogenesis processes and to improve the quality of embryos for ART. Since blastoids are a powerful platform for studying early implantation and embryo-endometrium interactions, blastoids create prospects for individualized medicine in ART. In addition, blastoids allow interspecies comparison of early embryogenesis, which is of particular importance for translating data from animal models to humans. All these possibilities make blastoids a universal model for fundamental research, drug testing, toxicology studies, and the development of new strategies in regenerative medicine and ART.

## Figures and Tables

**Table 1:** Evaluation of the relevance of using animal models for studying early human embryogenesis.

Species	Advantages	Limitations	Relevance to Humans
Mouse (*Mus musculus*)	Availability, genetically modified lines [71] Rapid development, high fertility [72]	ZGA at the 2-cell stage (earlier than in humans) [73] Cylindrical epiblast Difference in implantation mechanisms [74]	Low in morphology, average for genetic and molecular studies
Pig (*Sus scrofa*)	ZGA at the 4-cell stage (comparable to humans) [75] Large embryos, easy to manipulate [76]	Implantation is superficial and is accompanied by elongation of the embryo (not typical for humans) [77]	Medium, useful for testing environments and embryo metabolism
Cattle (*Bos taurus*)	ZGA at the 8–16 cell stage (comparable to humans) [78] Long preimplantation period (6–12 days) [79]	Implantation is superficial and is accompanied by elongation of the embryo (not typical for humans) [77]	High, especially for studying culture conditions and preimplantation development
Old world primates (*Macaca* spp.)	ZGA at 6–8 cell stage (comparable to humans) [80] Discoid epiblast Formation of amnion and hypoblast and gastrulation similar to humans [81]	Shorter development periods [81] Difficult maintenance, high costs [82] Ethical restrictions [83]	Very high, most relevant model among animals

## Data Availability

Data are contained within the article.

## Abbreviations

The following abbreviations are used in this manuscript:

ART	Assisted Reproductive Technologies
PSC	Pluripotent Stem Cell
SES	Synthetic Embryo Systems
ICM	Inner Cell Mass
TSC	Trophoblast Stem Cells
IVF	*In Vitro* Fertilization
ISSCR	International Society for Stem Cell Research
LIF	Leukemia Inhibitory Factor
IGF	Insulin-like Growth Factors
VEGF	Vascular Endothelial Growth Factor
CSF	Colony-Stimulating Factor
HB-EGF	Heparin-Binding Epidermal Growth Factor
PIF	Preimplantation Factor
MEA	Mouse Embryo Assay
ZGA	Zygotic Genome Activation
TLI	Time-Lapse Imaging
AI	Artificial Intelligence
PGT	Preimplantation genetic testing
PGT-A	Preimplantation Genetic Testing for Aneuploidy
PGT-M	Preimplantation Genetic Testing for Monogenic Disorders
mESC	Mouse Embryonic Stem Cells
iPSC	Induced Pluripotent Stem Cells
OKSM	OCT3/4, SOX2, KLF4, cMYC
hPSC	Human Pluripotent Stem Cell
EGF	Epidermal Growth Factor
VGLL1	Vestigial-like family member 1
TE	Trophectoderm
HYP	Hypoblast
PrE	Primitive Endoderm
EPSC	Extended Pluripotent Stem Cell
TGF-β	Transforming Growth Factor β
LPA	Lysophosphatidic acid
EPI	Epiblast
5iLAF	5 inhibitors + LIF + Activin A + FGF2
4CL	4-component culture with LIF
FGF	Fibroblast Growth Factor
